# Comparative Analysis of mRNA Targets for Human PUF-Family Proteins Suggests Extensive Interaction with the miRNA Regulatory System

**DOI:** 10.1371/journal.pone.0003164

**Published:** 2008-09-08

**Authors:** Alessia Galgano, Michael Forrer, Lukasz Jaskiewicz, Alexander Kanitz, Mihaela Zavolan, André P. Gerber

**Affiliations:** 1 Institute of Pharmaceutical Sciences, ETH Zurich, Zurich, Switzerland; 2 Biozentrum, University of Basel, Basel, Switzerland; Wellcome Trust Sanger Institute, United Kingdom

## Abstract

Genome-wide identification of mRNAs regulated by RNA-binding proteins is crucial to uncover post-transcriptional gene regulatory systems. The conserved PUF family RNA-binding proteins repress gene expression post-transcriptionally by binding to sequence elements in 3′-UTRs of mRNAs. Despite their well-studied implications for development and neurogenesis in metazoa, the mammalian PUF family members are only poorly characterized and mRNA targets are largely unknown. We have systematically identified the mRNAs associated with the two human PUF proteins, PUM1 and PUM2, by the recovery of endogenously formed ribonucleoprotein complexes and the analysis of associated RNAs with DNA microarrays. A largely overlapping set comprised of hundreds of mRNAs were reproducibly associated with the paralogous PUM proteins, many of them encoding functionally related proteins. A characteristic PUF-binding motif was highly enriched among PUM bound messages and validated with RNA pull-down experiments. Moreover, PUF motifs as well as surrounding sequences exhibit higher conservation in PUM bound messages as opposed to transcripts that were not found to be associated, suggesting that PUM function may be modulated by other factors that bind conserved elements. Strikingly, we found that PUF motifs are enriched around predicted miRNA binding sites and that high-confidence miRNA binding sites are significantly enriched in the 3′-UTRs of experimentally determined PUM1 and PUM2 targets, strongly suggesting an interaction of human PUM proteins with the miRNA regulatory system. Our work suggests extensive connections between the RBP and miRNA post-transcriptional regulatory systems and provides a framework for deciphering the molecular mechanism by which PUF proteins regulate their target mRNAs.

## Introduction

Gene expression is regulated at multiple levels to ensure coordinated synthesis of the cells' macromolecular components. Besides transcriptional regulation, it is becoming increasingly recognized that control of the post-transcriptional steps has substantial impact on gene expression with widespread physiological implications [1], [2]. This regulation is mediated by hundreds of RNA-binding proteins (RBPs) that are encoded in eukaryotic genomes and bind to sequence/structural elements in mRNAs, and thereby regulate the localization, translation or decay of messages [3]–[7]. On the other hand, microRNAs (miRNAs), ∼22 nucleotide (nt) long RNA molecules, can repress gene expression by base-pairing with sequences in 3′-untranslated regions (3′-UTRs) of messages and thus inhibit their translation or promote decay [8], [9].

The PUmilio-Fem-3-binding factor (PUF) proteins comprise an evolutionarily conserved family of RNA-binding proteins that are implicated in various physiological processes 10, 11. They are defined by the presence of an RNA-binding domain, termed Pumilio-homology domain (Pum-HD), which consists of eight repeats, each of which makes contact with a different RNA base [12]–[15]. PUF proteins bind to an RNA element that comprises a core ‘UGUR’ tetranucleotide followed by 3′-UTR sequences that vary among PUF proteins. In concert with other factors, PUFs repress gene expression by inhibiting translation or promoting decay [16], [17], [18].

The study of PUF proteins in diverse model organisms revealed widespread roles for these proteins in embryonic development, stem-cell maintenance and neurogenesis [10], [11]. In the fruit fly *Drosophila melanogaster*, Pumilio (Pum) is required for proper anterior/posterior patterning during early embryogenesis by repression of the translation of *hunchback* mRNA [19]. Furthermore, Pum is also involved in the development and migration of primordial germ cells [20], [21], [22], and it may be implicated in long-term memory formation and neuronal excitability [23], [24], [25]. In the nematode *Caenorhabditis elegans*, Fem-3 mRNA Binding Factors 1 and 2 (FBF-1, FBF-2) regulate the germline switch from spermatogenesis to oogenesis by repressing *fem-3* mRNA translation [26]. The six yeast *Saccharomyces cerevisiae* PUF proteins (Puf1p–Puf6p) regulate aging, mating-type switching and mitochondrial function [10], [27], [28].

Much less is known about the functions of PUF homologs in vertebrates. Two paralogous PUF proteins exist in human, termed Pumilio homolog 1 (PUM1) and Pumilio homolog 2 (PUM2). PUM1 and PUM2 are often co-expressed in diverse tissues suggesting that they may occasionally act redundantly [11], [29], [30]. Based on few studies investigating PUM2 function, it is assumed that mammalian PUFs have physiological roles analogous to the non-vertebrate homologs: in germ cells, PUM2 interacts with deleted in azoospermia (DAZ), DAZ-like (DAZL) proteins, and the meiotic regulator BOULE (BOL), which are RBPs that function in early germ line stem cells [29], [31]. Moreover, mouse *Pum2* mutants have smaller testes, although fertility seems not to be affected [32]. Based on these results, a role for *Pum2* in the maintenance of germline stem cells was proposed [29], [31]. PUM2 was recently found to negatively regulate the expression of MAPK1 (mitogen-activated protein kinase 1, ERK2) and MAPK14 (mitogen-activated protein kinase 14) in human embryonic stem cells and in the *C. elegans* germline. MAPK1 and MAPK14 are kinases acting in the MAPK/ERK pathway that represses stem cell self-renewal [33] and hence, these results sustain an ancestral role for PUF proteins in maintenance and self-renewal of stem cells [10]. Recent evidence suggests additional roles of mammalian PUM2 in neurons i.e. for maintaining synapse morphology and function [30], [34].

A major obstacle in the study of PUF proteins (and of RBPs in general) is the lack of knowledge about the specific mRNA targets. Systematic identification of the RNAs associated with RBPs *in vivo* is therefore needed to identify the potential RNA targets that may undergo regulation. In addition, identifying target RNAs of conserved RBPs in diverse organisms should provide insight into evolutionary aspects of post-transcriptional regulatory networks. We have previously identified the mRNA targets for PUF proteins in the yeast *Saccharomyces cerevisiae* and the fruit fly *Drosophila melanogaster*, revealing association of PUFs with distinct subsets of mRNAs encoding functionally or cytotopically related proteins that are part of the same macromolecular complex, localize to the same subcellular region or act in the same signal transduction pathway [35], [36]. For example, yeast Puf3p binds nearly exclusively to nuclear encoded mRNAs for mitochondrial proteins, whereas *Drosophila* Pum in ovaries of adult flies associates with mRNAs encoding nuclear proteins involved in nucleotide metabolism and transcriptional regulation, and many mRNAs coding for proteins localized to organelle membranes. These studies provided strong evidence for the presence of a highly organized post-transcriptional regulatory system that coordinates the fates of functionally related groups of mRNAs as ‘post-transcriptional operons’ or RNA regulons [2], [37], [38]. Moreover, the knowledge of RBP target RNAs initiated diverse follow-up experiments unraveling new functions of these proteins [25], [28], [39], [40].

We have now undertaken a systematic analysis of the mRNAs associated with the two human PUM proteins to provide a framework for the study of their functional implications. Surprisingly, our list of experimentally defined PUM targets predicts extensive connections to the miRNA regulatory system, providing a first indication that ‘cross-talk’ between translational regulation through RBPs and miRNAs may be more frequent than previously appreciated [41], [42], [43].

## Results

### Human PUM 1 and PUM 2 associate with hundreds of mRNAs in HeLa S3 cancer cells

To identify mRNAs associated with human PUM proteins, we used a modified Ribonucleoprotein-ImmunoPrecipitation Microarray (RIP-Chip) approach on HeLa S3 cancer cells that express both PUM1 and PUM2 (Figure S1A) [44]. PUM ribonucleoprotein (RNP) complexes were captured from cell-free extracts with specific antibodies coupled to either protein G (PUM1) or protein A (PUM2) sepharose beads, and then eluted with SDS-EDTA (Figure S1B). To control for non-specifically enriched RNAs, the same procedure was performed with beads that were not coupled with immunoprecipitating antibodies (mock samples). RNA was isolated from extracts (input) and from the immunopurified (IPed) samples, amplified, and labeled with Cy3 and Cy5 fluorescent dyes, respectively. The labeled RNA probes from total RNA and IPed RNA were mixed and competitively hybridized to human cDNA microarrays that contained probes for ∼26,000 transcripts. In this assay, the ratio of the two RNA populations at a given array element reflects the enrichment of the respective mRNA by the PUM affinity purification [35], [36].

**Figure 1:**
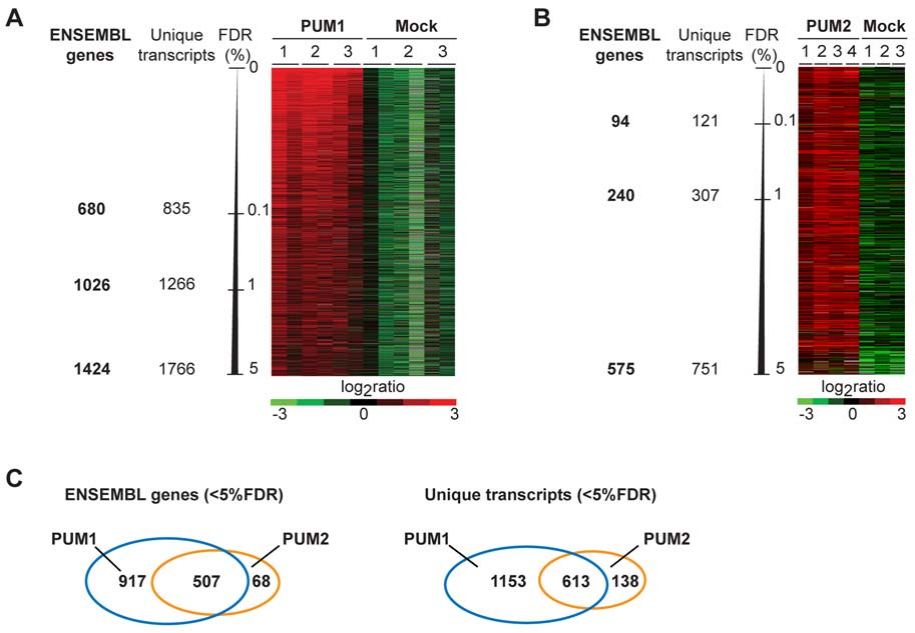
mRNAs specifically associated with human PUM proteins. Rows represent unique transcripts ordered according to increasing FDRs determined by SAM analysis. Columns represent individual experiments. The colour code indicates the degree of enrichment (green-red log_2_ ratio scale). (A) mRNAs associated with PUM1. Three experiments with PUM1 protein and three mock experiments both with dye-swap technical replicates are shown. (B) mRNAs associated with PUM2. Four experiments with PUM2 protein and three mock experiments are shown. (C). Venn diagram representing overlap between PUM1 and PUM2 targeted transcripts (right) and the corresponding genes (ENSEMBL, left).

To generate a list of mRNAs that were consistently enriched by PUMs and hence represent likely targets, we compared association of transcripts from PUM affinity isolations to the mock isolates by unpaired two-class Significance Analysis of Microarrays (SAM) and determined false discovery rates (FDRs) for each array element [45]. 1766 transcripts representing 1424 ENSEMBL annotated genes were consistently associated with PUM1 with FDRs of less than 5%. (Figure 1A, Table S1, a complete list of PUM1 mRNA targets is provided in Table S2). Likewise, we identified 751 transcripts (575 ENSEMBL genes) that were reproducibly associated with PUM2 with FDRs of less than 5% (Figure 1B, Table S1, a complete list of PUM2 mRNA targets is provided in Table S3). Strikingly, 507 (88%) of the PUM2 target genes were also among the experimentally defined PUM1 targets, indicating that the two human PUM paralogs have very similar substrate specificities and possibly act redundantly on common targets (Figure 1C). This finding correlates with the high amino acid conservation among PUM paralogs (83% similarity) and their respective RNA-binding domains (PUM-HD; 91% identity), where all of the critical amino-acids that directly contact RNA are fully conserved [13]. Furthermore, immunoblot analysis of PUM1 and PUM2 RIP eluates with α-PUM2 and α-PUM1 antibodies, respectively, did not show co-immunoprecipitation of the two paralogous proteins, thus excluding the possibility that the target overlap was simply due to simultaneous protein pull-down (Figure S1B).

In spite of the extensive overlap between the target sets of the two proteins, 138 PUM2 associated transcripts (representing 68 ENSEMBL annotated genes) did not pass the threshold to be selected as PUM1 target. Likewise, we identified over 1000 transcripts (representing 917 genes) that were only associated with PUM1 but not with PUM2 (Tables S2, S3). However, we observed substantial PUM2 protein degradation during the RIP procedure (Figure S1, data not shown) and hence, may have lost associations with a fraction of mRNA targets during the procedure, possibly reducing the number of identified targets. Apart from this, false-positives from unspecific antibody binding, or other PUM-interacting proteins that pulled down additional mRNAs could have contributed to differential mRNA associations. However, since most transcripts bear a canonical PUF-binding motif (see below), we believe that they represent true PUM targets. Differential associations may be attributed to slightly different substrate selectivity of the paralogous PUM proteins, possibly defined by additional sequence or structural elements in the vicinity of the PUF-binding site.

### Human PUM proteins associate with functionally related messages

To identify functional themes among the mRNAs associated with PUM1 and PUM2, we searched for shared Protein ANalysis THrough Evolutionary Relationships (PANTHER) [46] and Gene Ontology (GO) [47] annotations in the list of PUM1 and PUM2 mRNA targets with FDR<5% (Table 1, for a detailed list of significant annotations see Table S4). PANTHER pathway analysis of PUM1 targets revealed significant enrichment of components that regulate angiogenesis (*p*<8×10^−7^) or that mediate inflammatory/immune responses (T and B cell activation, *p*<5×10^−4^ and *p*<10^−2^, respectively). We also found strong enrichment of pathways important for cell-proliferation and stress response such as the Ras (*p*<1×10^−6^), the platelet-derived growth factor (PDGF, *p*<3×10^−4^) and epidermal growth factor (EGF, *p*<10^−2^) signaling pathways. Although several components of these pathways were also associated with PUM2, the respective terms did not reach statistical significance. The analysis for PUM2 targets revealed only two terms with weak statistical significance: the p53 pathway (*p*<10^−2^), which was also weakly enriched among PUM1 targets (p<10^−3^), and several messages coding for proteins related to Parkinson's disease (*p*<2×10^−2^) (Table 1, Table S4).

**Table 1:** Significantly shared PANTHER and GO annotations among PUM1 and PUM2 mRNA targets.

Category	Term	PUM1 *p*-value	PUM2 *p*-value
Pathway (PANTHER)	Angiogenesis	8×10^−7^	
	Ras Pathway	1×10^−6^	
	PDGF signaling pathway	3×10^−4^	
	T cell activation	5×10^−4^	
	p53 pathway	1×10^−3^	9×10^−3^
	Interleukin signaling pathway	1×10^−2^	
	EGF receptor signaling pathway	1×10^−2^	
	B cell activation	1×10^−2^	
	Parkinson's disease		2×10^−2^
Biological Process (PANTHER)	Nucleoside, nucleotide and nucleic acid metabolism	1×10^−19^	1×10^−6^
	Cell cycle	1×10^−14^	9×10^−7^
	mRNA transcription	3×10^−13^	5×10^−4^
	Protein phosphorylation	2×10^−8^	3×10^−2^
	Intracellular protein traffic	3×10^−7^	6×10^−3^
	Intracellular signaling cascade	6×10^−6^	3×10^−2^
	Cell proliferation and differentiation	5×10^−5^	
	Developmental processes	7×10^−5^	
	Oncogenesis	1×10^−4^	
	DNA repair	4×10^−4^	
	MAPKKK cascade	1×10^−2^	
Molecular Function (PANTHER)	Nucleic acid binding	6×10^−11^	4×10^−6^
	Transcription factor	2×10^−10^	
	Kinase	3×10^−9^	
	Non-receptor serine/threonine protein kinase	3×10^−7^	
	RNA-binding protein/mRNA binding	4×10^−4^	
	Membrane traffic protein		1×10^−2^
Component (GO)	Intracellular membrane-bound organelle	7×10^−62^	2×10^−25^
	Nucleus	6×10^−44^	1×10^−14^
	Cytoplasm	2×10^−36^	9×10^−12^
	Organelle lumen	4×10^−18^	3×10^−7^
	Nuclear lumen	2×10^−16^	8×10^−6^

PANTHER: total 25,431 NCBI annotated genes.

GO: total 35,541 EBI annotated genes.

**Figure 2:**
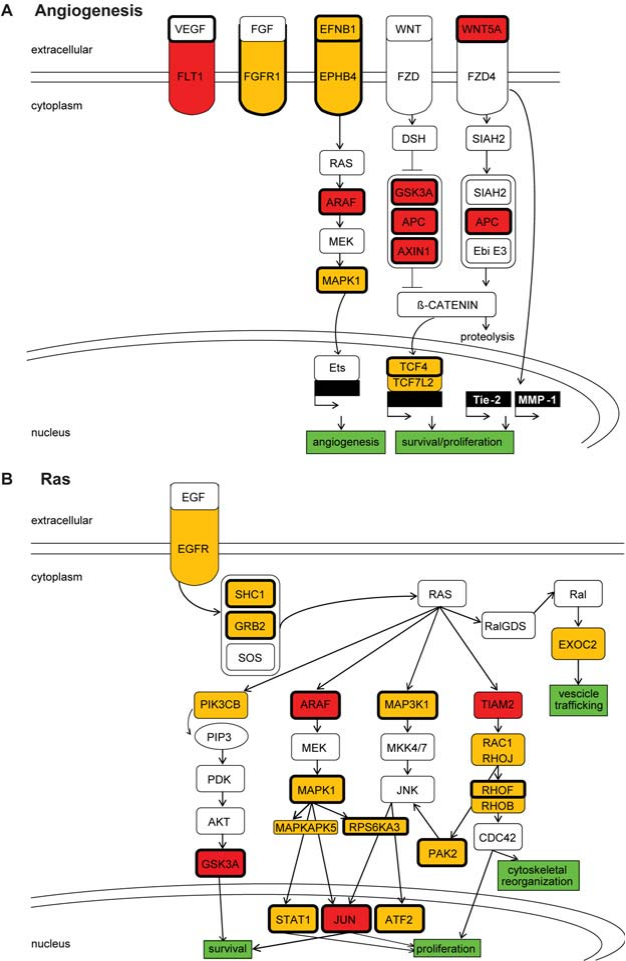
PUM targets encode proteins acting in cancer related pathways. Components whose mRNAs are associated with PUM1 are depicted in yellow, those bound by both PUM1 and PUM2 are shown in red. Messages that contain a PUF motif are shown with a thick black border. (A) Regulators of angiogenesis. PUM associated messages code for the tyrosine kinase receptors fms-related tyrosine kinase 1 (FLT1), which is the vascular endothelial growth factor receptor 1, fibroblast growth factor receptor 1 (FGFR1) and EPH receptor B4 (EPHB4) and its ligand ephrin-B1 (EFNB1). These receptors and their ligands can trigger signals that induce angiogenesis [73], [74], [75]. ARAF (v-raf murine sarcoma 3611 viral oncogene homolog) and MAPK1 (mitogen-activated protein kinase 1) are part of the RAS/RAF/MAPK pathway that can activate ETS (E26 transformation specific sequence) family transcription factors that promote angiogenesis [76]. Human PUM proteins commonly target messages of both canonical (Wnt/ß-catenin) and non-canonical (Wnt/calcium signaling and planar cell polarity) pathways: WNT5A (wingless-type MMTV integration site family, member 5A) activates non-canonical Wnt signaling [77], which induces proliferation of endothelial cells *in vitro*. WNT5A is thought to promote the expression of the angiogenic effectors MMP1 (matrix metallopeptidase 1), and TEK (endothelial TEK tyrosine kinase, TIE-2) [77]. PUM1 and PUM2 commonly target components of the “ß-catenin destruction complex” consisting of the serine/threonine kinase GSK3A (glycogen synthase kinase 3 alpha), which phosphorylates ß-catenin marking the protein for ubiquitylation and rapid degradation by the proteasome, the tumor suppressor APC (adenomatous polyposis coli), and the scaffold protein AXIN1. PUM1 further associates with mRNAs coding for the co-factors TCF/LEF1 (transcription factor/lymphoid enhancer-binding factor 1) that become activated when ß-catenin enters the nucleus. This includes TCF4 and TCF7L27 (transcription factors 4 and 7-like 2), that stimulate the transcription of genes implicated in cell growth regulation [50]. (B) Activators and effectors of RAS [49], [78]. PUM bound messages code for EGFR (epidermal growth factor receptor), adaptor proteins GR*B2* (growth factor receptor-bound protein 2) and SHC1 (Src homology 2 domain containing transforming protein 1), which activate Ras proteins upon recruitment of the guanine nucleotide exchange factor SOS (son of sevenless). RAS interacts specifically with ARAF, MAP3K1 (mitogen-activated protein kinase kinase kinase 1), PIK3CB (phoshoinositide-3-kinase, catalytic, beta polypeptide) and TIAM2 (T-cell lymphoma invasion and metastasis 2), which can initiate cascades of protein-protein interactions and further activate more specific signaling pathways. Components of the Raf/MEK/ERK and the MEKK/SEK/JNK pathways are covered by PUM1 targets encoding mitogen-activated protein kinases MAPK1 and MAPKAPK5 (mitogen-activated protein kinase-activated protein kinase 5), and RPS6AK3 (ribosomal protein S6 kinase, polypeptide 3). These pathways target the transcription factors JUN (jun oncogene), ATF2 (activating transcription factor 2) and STAT1 (signal transducer and activator of transcription), which commonly induce cell proliferation [49]. The PI3K-mediated (PIK3CB) signal is further triggered by activation of protein kinase B (AKT1, v-akt murine thymoma viral homolog 1) and phosphorylates GSK3A. PUM1 also targets effectors downstream of TIAM such as the GTP-binding protein RAC1 (ras-related C3 botulinum toxin substrate 1) and the Ras homologs gene family members B, F and J (RHOB, RHOF, RHOJ), which are all components of the TIAM/RAC/RHO signaling pathway implicated in the reorganization of the actin cytoskeleton [79]. The RAC effector PAK2 (p21 protein- activated kinase 2) is involved in cell migration and invasion [80], and EXOC2 (exocyst complex component 2) induces vescicle trafficking upon RAL (Ras-related) activation [49].

We were intrigued by the finding that PUM targets often encode proteins linked to angiogenesis - the process that promotes the formation of new blood vessels - and to the Ras (rat sarcoma) signaling pathway, which virtually affects every aspect of cell biology [48], [49]. We have therefore further mapped the interactions of the encoded proteins (Figure 2). Thirty-seven PUM1 bound mRNAs are assigned to the term ‘angiogenesis’ by PANTHER (Figure 2A). These include messages for diverse tyrosine kinase receptors including fms-related tyrosine kinase 1 (FLT1), which is a receptor for vascular endothelial growth factor A (VEGF A), a main inducer of angiogenesis. Even though VEGF A was not selected as a PUM target (FDR>86%), the 3′-UTR binds to PUM *in vitro* and bears a canonical PUF-binding motif, suggesting that PUM may regulate VEGF A expression (see below). Furthermore, PUM also targets components that transduce the intracellular signals downstream of these receptors and that are, at least in part, related to angiogenesis. For instance, parts of the wingless (Wnt) signaling pathway, including the three main components of the ‘ß-catenin destruction complex’ [50], or activators and effectors of Ras (Figure 2B) [49]. Finally, PUMs also bind diverse messages that are final targets of these signaling pathways, such as transcription factors that induce expression of angiogenic modulators or regulate cell proliferation or survival (Jun, STAT1, TCF4, TCF7L2). However, there is no apparent preference for PUMs to act selectively on positive or negative regulators of angiogenesis.

We finally searched for subcellular localization among PUM targets revealing that PUM associated mRNAs preferentially encode membrane-bound, cytoplasmic and nuclear proteins (Table 1, Table S4). The latter compartment mainly relates to transcription factors and their regulators, but also to RBPs. In this regard, PUM2 mRNAs was highly associated with PUM1 and PUM2 (FDR∼0), suggesting the presence of negative feed-back loops for self-regulation of PUM expression. In the cytoplasm, PUM1 targets many messages coding for kinases, in particular non-receptor serine/threonine protein kinases. Most of these messages cannot be found among the PUM2 associated mRNAs, indicating the presence of additional factors that direct the binding of functional groups of mRNAs to PUM proteins.

### Conservation of functional groups but not of homologous messages between yeast, fly and human

We have previously mapped the mRNAs associated with *Drosophila* Pum in adult flies, and we wondered whether these interactions may have been evolutionarily conserved [36]. We noticed partial overlap of functional groupings made of proteins encoded by PUF associated mRNAs. As seen for the human PUM proteins, *Drosophila* Pum preferentially targets messages coding for proteins located on membrane-bound organelle (*p*<10^−7^) and nuclear proteins (*p*<10^−5^), including transcription factors, cyclins and RNA-binding proteins [36]. We therefore asked whether this consistency is directly reflected by association of the homologous messages with the different PUF proteins. We retrieved human homologs for the 1090 *Drosophila* Pum and for the 220 yeast Puf3p mRNA targets. Notably, among yeast Puf proteins, Puf3p is most related to human PUM and targets messages for nuclear encoded mitochondrial proteins [35], a functional class that is not particularly enriched among human PUMs. More than 40% of the *Drosophila* and yeast Puf3p targets had an assigned human homolog - however, only a small fraction of these messages were also among our experimentally determined human PUM targets: 17% and ∼7% of *Drosophila* Pum and a similar fraction of Puf3p homologs were among PUM1 and PUM2 targets (Table S5). Therefore, the conservation of functional themes among targets in human and *Drosophila* is not directly reflected by the association with homologous messages. Moreover, this indicates that the suspected conservation of PUF's physiological functions may not necessarily imply the regulation of the same critical genes.

### A common and conserved sequence motif among PUMILIO mRNA targets

**Figure 3:**
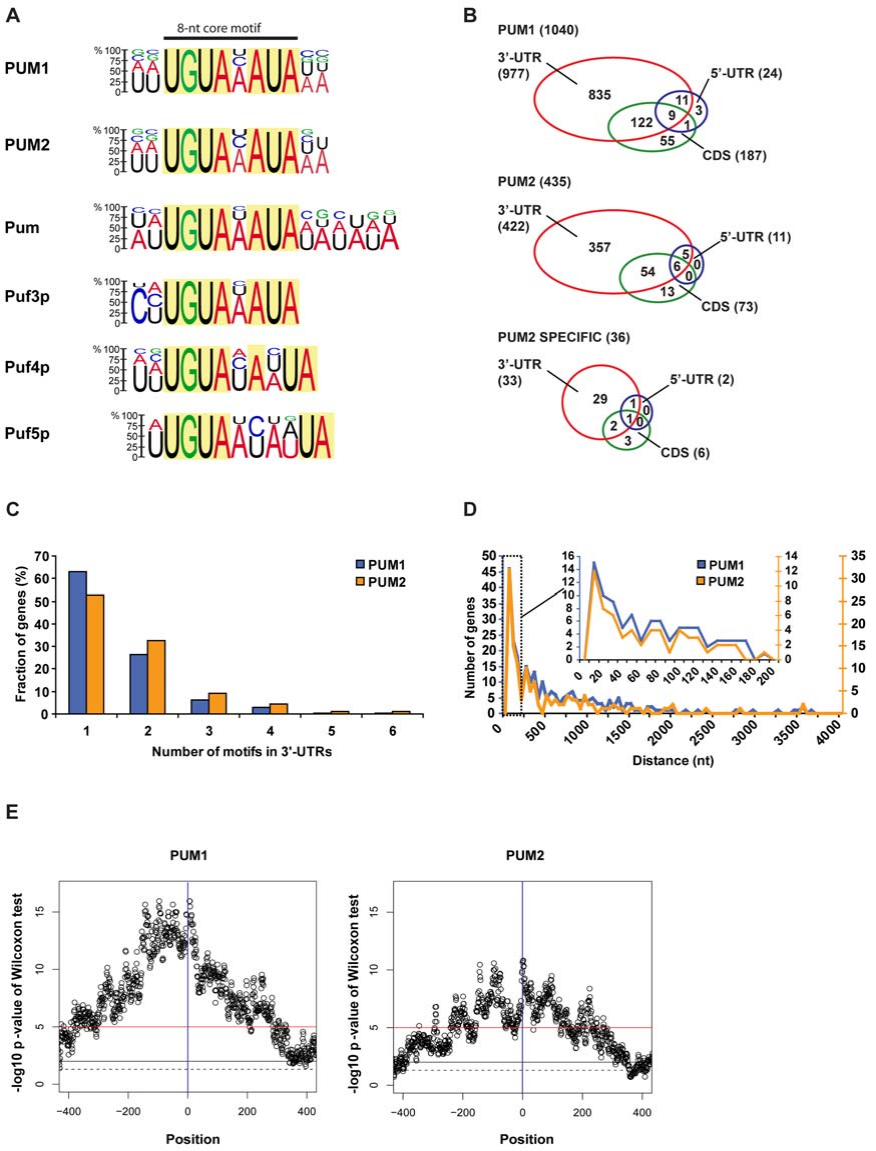
Analysis of an RNA consensus sequence associated with human PUM proteins. (A) PUF consensus motif in 3′-UTR sequences associated with PUM1, PUM2, *Drosophila* Pum and yeast Puf3, Puf4 and Puf5 proteins [35], [36]. Height of the letters indicates the probability of appearing at the position in the motif. Nucleotides with less than 10% appearance were omitted. (B) Distribution of PUF consensus motifs. (C) Number of PUF motifs in the 3′-UTRs of PUM bound messages. (D) Distances between double PUF motifs present in 3′-UTR. Represented bins are 50 nts (0–4000 nts distance) and 10 nts (0–200 nts distance). (E) Analysis of PUF motif conservation among PUM1 and PUM2 targets. The x-axis shows the position (relative to the middle of the PUF motif), and the y-axis shows the logarithm (base 10) of the *p*-value from the Wilcoxon test determining whether conservation scores come from the same distribution for PUM targets and non-targets. The vertical blue line is drawn at position 0 corresponding to the PUF motif. The dashed black line is drawn at a *p*-value of 0.05, the continuous black line at a *p*-value of 0.01, and the red line at a *p*-value of 10^−5^, which is the threshold for significance considering multiple testing.

Characteristic sequence motifs have been previously found in the 3′-UTRs of the mRNA targets of different PUF-family members [10], [33], [35], [36]. Thus, we examined the sets of mRNAs that associate with PUM1 and PUM2 for the presence of common motifs using Multiple Expectation maximization for Motif Elicitation (MEME) as an unbiased motif discovery tool [51]. We compiled one hundred available 3′-UTR sequences among the most highly enriched PUM1 and PUM2 mRNA targets, and MEME analysis identified a 12-nt consensus sequence encompassing a highly conserved 8-nt core motif UGUA(AUC)AUA (Figure 3A). The 8-nt consensus motif is highly related to the *Drosophila* Pum and yeast Puf3p mRNA binding site [35], [36], indicating the conservation of the recognition element during evolution despite the lack of conservation of the PUF targets among the considered species. We further evaluated the occurrence of this motif among PUM mRNA targets by searching UTRs and coding sequences (CDSs) for the presence of the 8-nt core motif UGUAnAUA using PatSearch [52]. 69% of the PUM1 mRNA targets (*p*<10^−100^) and 74% of the PUM2 targets (*p*<10^−100^) contained the consensus motif in the 3′-UTR, which represents a striking enrichment compared to its genome-wide occurrence in 3′-UTRs (20% of all ENSEMBL annotated genes; 22% of all genes for which data could be obtained for microarray cDNA probes). We also found the motif highly overrepresented in the CDS of mRNA targets (13% of PUM1 and PUM2 mRNA targets with *p* values of <10^−100^ and 10^−15^, respectively), but it is almost absent in 5′-UTRs (Table 2, for detailed statistics on motif occurrences see Tables S6, S7). These results are consistent with the observed enrichment of PUF-binding motifs in coding sequences of mRNAs targeted by yeast PUF proteins [35]. Moreover, the functionality of PUF motifs in CDS has recently been demonstrated for paralytic (*para*) mRNA, which codes for a sodium channel expressed in neurons of *Drosophila* larvae [25].

**Table 2:** Number of PUF consensus motifs found in human PUMILIO targets (FDR<5%).

	Search option	Sequences	Motifs (%)	*p*-value
**PUM1**	3′-UTR	1416	977 (69)	<10^−100^
	CDS	1418	187 (13)	<10^−100^
	5′-UTR	1390	24 (2)	5×10^−2^
**PUM2**	3′-UTR	571	422 (74)	<10^−100^
	CDS	572	73 (13)	6×10^−16^
	5′-UTR	558	11 (2)	7×10^−2^

We next analyzed the distribution of PUF consensus motifs. Approximately 85% of PUM1 and PUM2 mRNAs targets bear the motif exclusively in the 3′-UTRs, 3–5% (PUM2 and PUM1, respectively) solely in the CDS, and ∼17% bear the motif in both the CDS and 3′-UTRs (Figure 3B). Most of the PUM bound messages have only one PUF motif (Figure 3C). However, a substantial fraction – one third of the PUM2 bound messages (32%) - bears at least two consensus PUF-binding motifs in the 3′-UTRs. The distance between multiple motifs is similarly distributed among the PUM1 and PUM2 mRNA targets ranging up to 4000 nts with median distances of 324 nts and 230 nts for PUM1 and PUM2 targets, respectively (Figure 3D). Nevertheless, a large proportion of the double motifs are located within 200 nts (94, 36% PUM1 and 58, 25% PUM2), and a ‘peak’ was found at a distance of ∼20 nts, indicating that the two motifs are preferentially located in close proximity (Figure 3D, inlet). Such repetitive occurrence of PUF binding sites may affect RNA regulation: different sites could have different affinities for PUF binding leading to dose-dependent or allosteric regulation. Such a mode of regulation has been proposed for messages of *C. elegans* MAP Kinase 1 (*mpk-1*) mRNA, which bears two distinct FBF binding sites with five-fold different binding affinities [33].

We finally questioned whether the positions within and around the PUF-binding motifs were evolutionarily conserved in mammals [53]. We used as measure of evolutionary conservation the phastCons score [54] representing the probability that a given nucleotide is part of a block of conservation, given the genome alignments of a number of placental mammals (human, chimpanzee, rhesus monkey, bush baby, treeshrew, rat, mouse, guinea pig, rabbit, shrew, hedgehog, dog, cat, horse, cow, armadillo, elephant and tenrec). In this way, we identified the PUF motifs in the PUM1 and PUM2 IPed transcripts (targets) and in the expressed transcripts that were not IPed (non-targets), and we used transcript-to-genome alignments to determine the genomic coordinates of the PUF motifs. For each nucleotide in the PUF motif and each nucleotide up to −400 nts upstream and to +400 nts downstream of the motif, we extracted the phastCons score. We then used the Wilcoxon test to determine whether the positions in and around PUF sites from IPed transcripts were more highly conserved than positions in and around non-IPed transcripts. The profiles of the Wilcoxon test for PUM1 and PUM2 sites, as represented by the logarithms of the *p*-values, are shown in Figure 3E. Position of PUF motifs in PUM1 and PUM2 targets are more conserved than in non-targets (*p*-values are smallest for positions within the PUM sites). Moreover, we found that the PUF motifs in PUM1 and PUM2 targets reside in longer (400 nucleotides) blocks of conservation compared to PUF motifs in non-targets. Thus, the observed constraints on the positions of PUF motifs in the PUM target mRNAs, but not in non-target RNAs suggests that these motifs are indeed functionally conserved. These findings further indicate that other factors may contribute to or modulate the functionality of PUM binding sites, for example recognition elements for cofactors like Nanos, which is known to interact with Pumilio to mediate translational repression [55].

### RNA pull-down experiments confirm PUM binding to selected substrates

**Figure 4:**
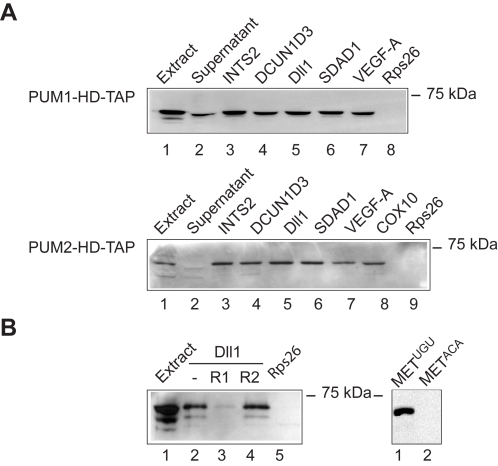
Validation of human PUM mRNA targets. RNA-protein complexes formed between biotinylated 3′-UTRs and extracts of HeLa S3 cells expressing PUM1-HD-TAP and PUM2-HD-TAP were purified on streptavidin magnetic beads and monitored for the presence of TAP-PUM-HD by immunoblot analysis with anti-PAP antibody. (A) Biotin-labeled 3′-UTR sequences for indicated genes (lanes 3 to 8) were incubated with PUM1-HD-TAP and PUM2-HD-TAP extracts (lane 1). Rps26 3′-UTR was used as negative control probe RNA (lanes 8/9). The supernatant after pull-down with INTS2 is shown in lane 2. (B) Validation of the PUF-binding motif. Biotinylated RNA corresponding to the Dll1 3′-UTR was combined with PUM1-HD-TAP extract (lane 2) and 100-fold excess of competitor RNA (R1; AUUGUAAAUA; lane 3) or control RNA where the core motif is mutated (R2; AUACAAAAUA; lane 4). A fragment of MET 3′-UTR bearing wild type (UGU) or mutant (ACA) PUF binding sites is shown in lanes 5 and 6.

To evaluate some of our identified PUM mRNA substrates, we performed RNA pull-down experiments using *in vitro* transcribed biotinylated mRNAs added to extracts prepared from HeLa cells expressing TAP-tagged PUM1-HD or PUM2-HD. We tested biotinylated 3′-UTR sequences of six potential targets that contain the PUF motif: integrator complex subunit 2 (INTS2), defective in cullin neddylation 1, domain containing 3 (DCUN1D*3*), delta-like 1 (Dll1), SDA1 domain containing 1 (SDAD1), VEGFA and hepatocyte growth factor receptor (MET). INTS2, MET and other members of the DCUN1 (DCUN1D1, DCUN1D4) and Dll gene families (Dll3) were among our list of IPed PUM mRNAs targets, whereas SDAD1 and VEGFA were not among the IPed messages, though they bear a conserved PUF binding motif. Moreover, SDAD1 was previously found to interact with PUM2 [56]. We also tested yeast cytochrome c oxidase (*COX10*), a known target for the yeast PUF3 protein, which bears the 8-nt core consensus motif [35], and a negative control RNA (Ribosomal protein S26, RpS26) that does not bind to PUFs [36]. All of the seven potential target mRNAs bound to both PUM1-HD and PUM2-HD, whereas the RpS26 control 3′-UTR sequence did not (Figure 4A). Moreover, addition of a 10-nt competitor RNA comprising the consensus sequence prevented binding to biotinylated Dll1 RNA, but no such competition was seen with a control RNA, in which the conserved UGU trinucleotide within the core was mutated to ACA (Figure 4B, data not shown). Likewise, mutation of this PUM binding site in a fragment of the MET RNA also abolished binding (Figure 4B). Notably, probing of the same immunoblots with PUM1 and PUM2 specific antibodies to detect the full-length proteins gave analogous results (data not shown). These results suggest that PUM1 and PUM2 have identical basal substrate specificities, reminiscent of the largely overlapping set of PUM1 and PUM2 mRNA targets identified by RIP-Chip. This suggests that the presence of the computationally inferred core motif is sufficient for association with human PUM proteins *in vitro*. However, since SDAD1 and VEGFA were not among our IPed mRNA targets, the *in vitro* binding activities may not always reflect *in vivo* association, which may be controlled by additional factors. The discrepancy may also be due to technical issues related to the experimental procedure, or the computational analysis of the microarray data.

### The PUF motif is enriched around predicted miRNA binding sites

**Figure 5:**
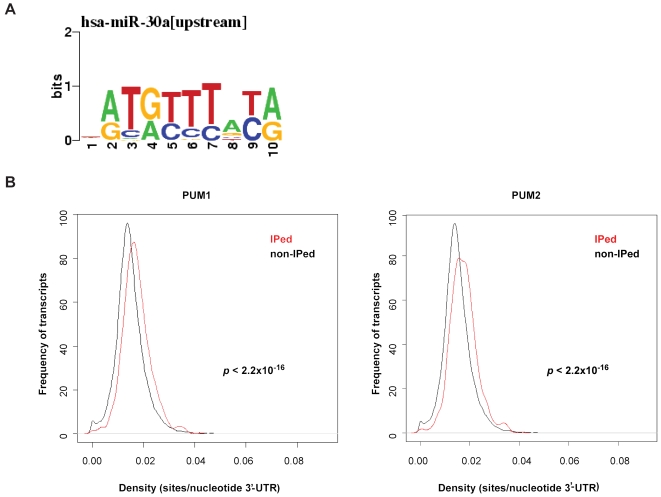
miRNA binding sites are enriched among human PUM targets. (A) Example of a motif identified using the Phylogibbs motif finding algorithm in the vicinity (400 nucleotides upstream) of high-confidence miR-30a target sites. The x-axis indicates the position of a nucleotide in the inferred motif, and the y-axis gives the information score (bits) at that position. The height of each letter is proportional to the frequency of the respective nucleotide at that particular position in the alignment of inferred sites. (B) Distribution of the density of high-confidence miRNA sites (sites per nucleotide of 3′-UTR) in the 3′ UTRs of PUM targets (IPed, red) and non-targets (non-IPed, black).

Initial application of the Phylogibbs algorithm for motif finding [57] to 3′-UTR regions around high-confidence predicted microRNA (miRNA) target sites [58] suggested that the PUF-binding motif could be enriched in these regions, as shown in Figure 5A (Zavolan, unpublished). However, this motif (UGUAnAUA) is A/U-rich, and high-confidence miRNA sites are known to reside in A/U-rich regions [58], [59]. Thus, we decided to test directly whether the PUF-binding motif indeed occurs in the vicinity of high-confidence miRNA sites at a higher frequency than expected at a random distribution, particularly given its nucleotide composition. We extracted from our miRNA target predictions [58] the top 1000 target sites with the highest probability of being under evolutionary selection, and an equal number of target sites with the lowest probability of being under evolutionary selection, by choosing for each miRNA having at least one high-probability target site, an equal number of low-probability sites. We then extracted 400 nucleotides upstream or 400 nucleotides downstream of the miRNA seed match (match to the nucleotides 1–7, 2–8, or 1–8 of the miRNA), and counted how many of the 1000 sequences contained the PUF consensus motif. For the upstream regions, we found 132 positive sequences (with high-probability miRNA sites) and 71 negative sequences (with low-probability miRNA sites) containing the PUF motif, whereas for the downstream regions, 159 positive sequences and 56 negative sequences contained the PUF motif (Table S8). This indicates that the frequency of the PUF motif is significantly higher in the environment of high-probability miRNA sites (*p*-values of 8.9×10^−6^ for the upstream and 1.8×10^−13^ for the downstream regions in the chi-square test). To rule out the possibility that this enrichment was simply due to the structure of the PUF motif, we performed the same analysis for all the 16384 possible motifs of the same structure as the PUF motif (i.e. first four bases defined, the fifth any of A/C/G/U and then the next three bases defined). Table S8 shows these results for all of the motifs for which the frequency was higher in regions around high-probability sites compared to regions around low-probability sites. As expected, we found that the environment of high-probability miRNA target sites is enriched in many A/U-rich elements. Strikingly, the PUF-binding motif is the second most significantly enriched motif (out of 6750 motifs) in the downstream regions of miRNA sites, and the fortieth most enriched motif (out of 6906 motifs) in the upstream regions. This test thus supports the hypothesis that the pumilio proteins share targets with the miRNA pathway.

As we mentioned above, the PUF motif is A/U-rich. We therefore wondered whether the enrichment that we observed was simply due to spurious matches to the PUF consensus that occur in the A/U-rich regions around high-confidence miRNA target sites. To test this, we generated by sequence shuffling 100 randomized sets of sequences with the same nucleotide composition as the regions around high-probability and low-probability miRNA target sites, respectively. We then counted the number of randomized sequences containing the PUF motif and performed the chi-square test. For the downstream regions, the lowest *p*-value that we observed in a randomized set was 10^−4^, much higher than 1.8×10^−13^ observed for the real data set. For comparison, the lowest *p*-value that we observed in a randomized set for the motif that was most enriched in the real data set (TTTTNTAA, *p* = 1.3×10^−14^) was 1.4×10^−10^. For the upstream regions the *p*-value of the real data set was only marginally lower compared to the lowest *p*-value we obtained for the randomized variants (8.9×10^−6^ compared to 5.1×10^−5^). These results indicate that the frequent occurrence of the PUF motif downstream of the high-confidence target sites cannot be explained simply by the nucleotide composition of these regions, and thus could suggest a functionally-relevant localization of the PUF-binding motif downstream of the miRNA sites for the interplay between the two systems.

### High-confidence miRNA binding sites are enriched in the 3′-UTRs of experimentally determined PUM targets

We wondered whether our experimentally determined sets of PUM targets provide evidence that miRNAs and PUMs share target mRNAs. Thus, we first selected from our experimental data sets PUM1 or PUM2 targets (IPed), as well as expressed transcripts that were not PUM1 and PUM2 targets (non-IPed). We then computed the density of high-probability miRNA sites (*p*≥0.5 computed by the method of Gaidatzis [58] (http://www.mirz.unibas.ch/ElMMo2) in the two data sets. The distribution of densities for IPed and not IPed transcripts is shown in Figure 5B. The *p*-value of the Wilcoxon test was <2.2×10^−16^ for both PUM1 and PUM2 targets, indicating that PUMs tends to target transcripts that are enriched in high-probability miRNA sites, and suggesting that there could be cross-talk between the two systems. In fact, evidence for an interaction of a PUF protein with the miRNA pathway already exists: it has been previously shown that *C. elegans puf-9* is required for the repression of the *let-7* miRNA target HunchBack Like (*hbl-1*) [43]. We therefore selected a set of candidates that appear most promising for follow-up studies. These are 197 PUM1 and 77 PUM2 targets that contain a high-probability miRNA site and a PUF site conserved among human, rhesus, cow, dog and mouse that are located within 50 nucleotides of each other (Table S9).

## Discussion

We have systematically analyzed the mRNAs associated with the two human Pumilio RNA-binding proteins, PUM1 and PUM2 in HeLa S3 cancer cells, using a method that combines the recovery of endogenous RNP complexes and DNA microarray analysis of the associated mRNAs [2], [44], [60], [61], [62]. We identified more than one thousand PUM1 and hundreds of PUM2 associated mRNAs, providing the first comparative analysis of mRNAs associated with paralogous PUF proteins in vertebrates. Our data suggests that PUM proteins potentially regulate approximately 15% of the cell's transcriptome. A similar fraction of the transcriptome was found to be associated with the five yeast PUF proteins and the *Drosophila* homolog Pumilio, indicating that PUF proteins generally coordinate large sets of mRNAs with functional implications that may not be simply attributed to a few specific mRNA targets.

The sets of human PUM1 and PUM2 associated mRNAs strongly overlapped, suggesting that PUM1 and PUM2 have similar substrate specificities (Figure 1). The presence of identical PUF-binding elements among the PUM1 and PUM2 associated mRNAs (Figure 3), and equal binding of PUM1 and PUM2 to a set of synthetic RNAs in RNA pull-down experiments further corroborated this notion (Figure 4). These results agree with recent structural studies of PUM-HD in complex with RNA. The PUM-HDs of mammalian PUM proteins are highly related (91% amino acid identity) and all of the critical amino-acids that make direct contact with the RNA are fully-conserved [11]. The human PUM proteins are therefore different from the PUF proteins in *S. cerevisiae* or *C. elegans*, where individual PUF family members have altered substrate specificities and only marginally share common sets of mRNAs [35], [63]. However, despite this large overlap of mRNA targets in HeLa cells, each PUM was also associated with a distinct set of mRNAs indicating that additional factors may further specify substrate selectivity *in vivo*.

Functionally related groups of mRNAs were often associated with both PUM1 and PUM2 (Table 1, Figure 2). However, some of them were preferentially enriched with either PUM1 or PUM2. For instance, angiogenesis-related proteins were mainly enriched among PUM1 targets whereas the transcripts coding for proteins linked to Parkinson's disease were solely enriched among PUM2 targets. Notably, the same functional attributes can often be found among all genes with a conserved PUF motif in 3′-UTRs [53], including angiogenesis (*p*<10^−13^) and Parkinson's disease (*p*>10^−5^) (for a list of PANTHER and GO terms that are enriched among predicted PUF targets see Table S10). However, some functional groups were differentially enriched among experimentally determined and predicted mRNA targets: for example, the Ras signaling pathway was enriched among PUM1 targets, but not among the predicted ones, whereas the Wnt signaling pathway is only significantly overrepresented among the predicted targets (*p*<3×10^−8^). Moreover, a fraction of the predicted targets encode proteins involved in neurogenesis (p<2×10^−35^) possibly relating to PUM functions in neurons [23], [24], [25]. However, since we have analyzed PUM targets in cancer cells, these neuron-specific mRNA targets were not expected to be identified. In conclusion, these analyses revealed PUM- and possibly cell/tissue-specific functional attributes among the potentially regulated messages, and it will be a future challenge to investigate the functional roles of PUM regulation on key targets.

During preparation of this manuscript, a ribonomic analysis has been published where mRNAs associated with PUM1 were identified and analyzed [61]. This study by Morris *et al.* applied a very similar RIP-Chip approach as we did by using the same PUM1 antibodies on HeLa S3 cells. Morris *et al.* defined 726 PUM1 mRNA targets (representing 11.1% of the 6,539 expressed genes). 397 of these mRNA targets (55%) were also among our experimentally identified PUM1 targets with a 5% FDR; and for 902 of our defined PUM1 targets that were represented on their arrays, 756 (85%) were more enriched than the median IP enrichment (t-scores) of all mRNAs. Furthermore, Morris *et al.* also identified the core PUF motif in almost half of 3′-UTRs of mRNA targets. Therefore, our data is in broad general agreement with the data from Morris *et al.* despite some significant differences in the experimental set-up and microarray data analysis. For instance, different number of replicate arrays were used (three by Morris *et al. vs.* six in our study), different types of arrays and hybridization conditions (separate *vs.* competitive hybridization, total IP-ed RNA *vs.* amplified mRNA and oligo- *vs.* cDNA-arrays) and different statistical analyses (Gaussian mixture modeling with log of odds (LOD) scores *vs.* SAM). For instance, the larger number of replicates used in our study, our RNA amplification strategy and microarray analysis of more transcripts has probably lead to the identification of almost twice the number of mRNA targets compared to Morris *et al.* (1424 vs. 726) - most of them (>80%) bearing a PUM motif in the 3′-UTR or coding sequence. Nevertheless, both studies found that PUM1 associated mRNAs belong to a relatively small number of functional groups, mainly genes coding for proteins that function in transcriptional regulation and cell cycle/proliferation. These and our own results therefore strongly support the ‘RNA operon/regulon model’, which suggests the coordinate *cis*-/*trans*-regulation of multiple mRNAs coding for proteins with related functions [37], [38].

Interestingly, some functional groups have apparently been conserved between human and *Drosophila*. For instance, in both *Drosophila* and human, PUFs preferentially target messages for nuclear proteins that encode transcription factors and membrane associated proteins. However, it is intriguing that the conservation of functional themes among targets in human and *Drosophila* is not reflected by conservation of the particular homologous messages, which is consistent with data obtained by Keene and his colleagues [61]. This finding is intriguing in respect of the assumed conservation of physiological function of PUM proteins for germ-cell development and neurogenesis, suggesting that analogous phenotypes may be accomplished by targeting related mRNAs that are part of the same regulatory network. However, we want to note that this comparative analysis of targets in flies and human is hampered by the fact that PUM targets have been analyzed in different experimental set-ups (whole flies versus cultured cells) and therefore, the data is not directly comparable.

As seen in previous systematic analyses of mRNA targets of the yeast and *Drosophila* Pumilio proteins [35], [36], most of the human PUM targets contain a characteristic PUF-binding motif in the 3′-UTR, and a significant number of targets bear the motif in the CDS (Table 2). Moreover, almost half of the experimentally determined targets have multiple PUF binding motifs (Figure 3C). These findings raise the question about possible roles for the position and multiplicity of PUF motif in transcripts. The different binding sites may be used alternatively, or may bear different affinities as observed for the *C. elegans* FBF-1 and FBF-2 target *mpk-1* [33]. PUM1 and PUM2 may therefore compete or synergistically act on common RNA substrates. Slightly different preferences for RNA-binding, but also in the expression levels of PUM proteins may influence binding with alternative outcomes for the fate of the mRNA. Finally, it is possible that other factors contribute to or modulate the functionality of PUM binding sites. Actually, our analysis shows that not only the PUF sites are conserved among the PUM targets compared to the non-targets, but also longer blocks upstream and downstream of the PUF binding site showed significant conservation, suggesting that these could represent recognition sites for cofactors like nanos [55].

Our work provides first evidence that the PUF motif is enriched around predicted miRNA binding, offering the possibility for functionally relevant localization of the PUF binding site downstream the miRNA sites for the interplay between the two systems. This hypothesis is further sustained by the finding that high-confidence miRNA binding sites are significantly enriched in the 3′-UTRs of experimentally determined PUM1 and PUM2 targets. One example for interaction of PUF proteins with the miRNA pathway has already been described in *C. elegans*, where *puf-9* is required for repression of *hbl-1* by *let-7* miRNA [43]. The 3′-UTR of *hbl-1* transcript contains PUF binding sites as well as binding sites for the *let-7* miRNA family suggesting that PUFs and miRNAs cooperate to negatively regulate common targets [43]. On the other hand, it has also been observed that RBPs and miRNAs may directly compete with each other. For instance, the evolutionarily conserved RBP dead end homolog 1 (DND1) relieves miRNA-specific repression of several messages by binding to uridine-rich regions (URRs) which are located in close proximity to miRNA binding sites in the 3′-UTR, and thereby, prohibits miRNAs from associating with their target sites [42]. Another example constitutes the AU-rich element (ARE) binding protein Hu antigen R (ELAVL1) that counteracts *hsa-miR-122* mediated repression of a cationic amino acid transporter (SLC7A1, CAT-1) after stress treatment [41], [64]. Additional scenarios for how miRNAs could modulate RBP binding and function in a dynamic manner have also been hypothesized [65]. For instance, miRNA binding could alter the structure of the mRNA, which either ablates or provides binding sites for specific RBPs and further alters the fate of the mRNA target. Therefore, the functional interactions between PUF and miRNAs may well be very mRNA target-specific because many additional factors and combinatorial binding of RBPs and miRNAs may have an impact on its final fait. It will be the topic of future investigation to determine how PUF proteins interact with miRNAs on specific model substrates.

## Materials and Methods

### Oligonucleotide primers

For a list of primers see Supporting Text S1.

### Plasmid construction

Sequences coding for the C-terminal tandem affinity purification (TAP)-tag were amplified with primers TAP1-NotIFw and TAP2-XhoIRev from plasmid pBS1479 [66] by PCR, and cloned into pcDNA3.1 (Invitrogen) via NotI and XhoI restriction sites, generating plasmid pcDNA3.1-TAP. The sequences encoding the C-terminal part of PUM1 (AF315592; amino acids 746–1186) and PUM2 (AF31559; amino acids 624–1064) were PCR amplified from cDNA clones IRAUp969B1150D (PUM1) and IRAUp969G0177D (PUM2) from the Deutsches Ressourcenzentrum für Genomforschung (RZPD) with primer pairs PUM1-HD-EcoRVFw/PUM1-HD-NotIRev, and PUM2-HD-EcoRVFw/PUM2-HD-NotIRev, and cloned via EcoRV and NotI sites into pcDNA3.1-TAP, producing the plasmids pcDNA3.1-PUM1-HD-TAP and pcDNA3.1-PUM2-HD-TAP, respectively.

### Immunoblot analysis and antibodies

Protein samples were resolved on 8% SDS polyacrylamide gels and transferred to nitrocellulose membranes (BioRad). Membranes were blocked in phosphate buffered saline-0.1% Tween-20 (PBST) at 4°C overnight containing 5% low fat milk, probed with the designated specific antibodies and horse radish peroxidase (HRP)-coupled secondary antibodies, and developed with the enhanced chemiluminescence detection kit (Amersham). The following antibodies were used in this study (dilution indicated in brackets): goat anti-PUMILIO 1 (1∶25,000; Bethyl Laboratories, #300-201A), rabbit anti-PUMILIO 2 (1∶2,500; Bethyl Laboratories, #A300-202A); mouse anti-ß-actin (1∶3000; Sigma), HRP-linked anti-mouse (1∶2000; Sigma), HRP-linked anti-goat (1∶5000; Sigma); HRP-linked anti-rabbit (1∶5000; Amersham). HRP-coupled peroxidase anti-peroxidase antibody (PAP; 1∶5000; Sigma) was used to detect TAP-tagged proteins.

### Cell culture and transfections

HeLa S3 cells were grown in Dulbecco's Modified Eagle's Medium (DMEM) supplemented with 10% FBS (Gibco) and 1% penicillin/streptomycin (Gibco). The cells were grown in dishes (Falcon) in a humidified incubator at 37°C and 5% CO_2_. Two µg of PUM-HD expression plasmids were transfected into one million HeLa S3 cells with Superfect Transfection Reagent (Qiagen). Stable cell lines expressing PUM2-HD-TAP were obtained upon G418 antibiotic selection (400 µg/ml; Invitrogen).

### Ribonucleoprotein-ImmunoPrecipitation (RIP)

RNA affinity isolations were performed essentially as described [44]. HeLa S3 cells were grown in 15 cm dishes (Falcon) until 90% confluency, washed in PBS and collected by centrifugation at 3,000 *g* and 4°C for 5 min. Cells were resuspended in an equal volume of polysome lysis buffer (10 mM HEPES-KOH [pH 7.0], 100 mM KCl, 5 mM MgCl_2_, 25 mM EDTA, 0.5% IGEPAL, 2 mM dithiothreitol [DTT], 0.2 mg/ml Heparin, 50 U/ml RNase OUT™ [Invitrogen], 50 U/ml Superase IN™ [Ambion], 1× complete protease inhibitor tablet [Roche]) and lysed by repeated pipetting up and down. The suspension was centrifuged three times at 14,000 *g* at 4°C for 10 min and aliquots were in liquid nitrogen and stored at −80°C until use. Protein concentration was determined by the Bradford method (Bio-Rad protein assay, BioRad) with bovine serum albumin (BSA) as reference standard.

50 µl protein G or protein A sepharose beads (Amersham) were equilibrated in NT2 buffer (50 mM Tris-HCl [pH 7.5], 150 mM NaCl, 1 mM MgCl_2_, 0.05% IGEPAL) supplemented with 5% BSA (Equitech Bio), 0.02% sodium azide and 0.02 mg/ml heparin. 20 µg of goat anti-PUM1 and 50 µg of rabbit anti-PUM2 antibodies were then coupled to the blocked protein G and protein A beads, respectively, which were further incubated on a rotator for 12 hours at 4°C. No antibodies were added in mock control experiments. The beads were subsequently washed three times in NT2 buffer and resuspended in 5–10 ml NT2 buffer supplemented with 30 mM EDTA (pH 8.0), 1 mM DTT, 50 U/ml RNase OUT™ and 50 U/ml Superase IN™ (to decrease unspecific binding to the beads, NT2 buffer corresponding to ten volumes of extract was used). HeLa cell extract (20 mg protein) was added to the antibody-coupled or mock beads, which were then mixed on a rotator for 6 hours at 4°C. The beads were then thoroughly washed four times in ice-cold NT2 buffer and RNP complexes were eluted twice with 500 µl SDS-EDTA (50 mM Tris [pH 8.0], 100 mM NaCl, 10 mM EDTA, 1% SDS) for 10 min at 65°C.

### RNA isolation, amplification and fluorescent labeling

Total RNA was isolated from cell extracts and immunopurified samples with the *mir*Vana™ PARIS™ kit (Ambion). RNA was quantified with a NanoDrop device (Witeg AG). Poly-adenylated RNAs were amplified in the presence of aminoallyl-UTP with Amino Allyl MessageAmp II aRNA kit (Ambion). For this purpose, 500 ng total RNA from extracts and half (50–100 ng) of the immunopurified RNAs were used for amplification. 8 µg of the amplified RNAs (aaRNA) were fluorescently labeled with NHS-monoester Cy3 and Cy5 dyes (GE HealthSciences), except for mock RNA samples, where an aaRNA amount proportional to the yield obtained from corresponding PUM affinity isolates was used. For PUM1 RIPs, we performed three biological replicates with technical (dye swap) replicates (total six arrays). For PUM2 RIPs, we performed four biological replicates but omitted the dye swaps due to the lower aaRNA obtained after amplification (∼10 µg aaRNA from PUM2 RIPs, ∼40 µg aaRNA from PUM1 RIPs, ∼9 µg aaRNA from mock RIPs). The Cy3- and Cy5-labeled aaRNA samples were mixed and hybridized to human cDNA microarrays.

### Microarray analysis and data selection

Detailed methods for microarray experiments are available at http://cmgm.stanford.edu/pbrown/protocols/index.html. cDNA microarrays were produced by the Stanford Functional Genomic Facility and contained 43,197 human probes representing 26,524 Unigene cluster IDs (12,466 ENSEMBL annotated genes) spotted on Corning Ultra GAPS slides. Spotted cDNAs were cross-linked with 65 mJ of UV irradiation on slides, which were then post-processed for 1 hour at 42°C in pre-hybridization solution (5× SSC, 0.1% SDS, 0.1 mg/ml BSA), washed twice in 400 ml of 0.1× SSC for 5 min, dunked in 400 ml ultrapure water for 30 sec, and dried by centrifugation at 550 rpm for 5 min. Slides were used the same day.

Cy3- and Cy5-labeled aaRNA probes were mixed and applied to arrays in hybridization solution (3× SSC, 20 µg poly(A) RNA [Invitrogen], 20 µg yeast tRNA [Invitrogen], 20 µg Human Cot-1 DNA [Invitrogen], 20 mM HEPES [pH 7.0] and 0.3% SDS) for 18 h at 65°C. The arrays were then washed sequentially in 400 ml of 2× SSC with 0.1% SDS, 1× SSC, and 0.2× SSC. The first wash was performed for 5 min at 65°C, the subsequent washes were performed for 5 min at RT. The arrays were dried by centrifugation and immediately scanned with an AxonScanner 4200A (Molecular Devices). Data were collected using GENEPIX 5.1 (Molecular Devices). Arrays were normalized computationally by the Stanford Microarray Database (SMD) [67]. The data were filtered for signal over background of greater than 1.5 in the channel measuring aaRNA from extract, and only features that met these criteria in >50% of the arrays were included for further analysis. Log_2_ median ratios were retrieved and exported into Microsoft Excel.

To identify transcripts that were specifically enriched by association with PUM1 and PUM2, we performed two class Significance Analysis of Microarrays (SAM) on median centered arrays [45]. Comparing six arrays representing PUM1 affinity isolations (three independent experiments, each with a dye-swap replicate) with six arrays representing mock isolates (three independent experiments with dye swaps) identified 1674 transcripts representing 1266 annotated genes with FDRs<1% and 2196 transcript (1755 annotated genes) with FDRs<5% (Table S1; a list of PUM1 mRNA targets is shown in Table S2). Likewise, comparing four arrays representing independent PUM2 affinity isolations with three mock control arrays identified 400 transcripts (307 annotated genes) with FDR<1%, and 889 transcripts (751 genes) with FDRs<5% (Table S1; a list of PUM2 targets is shown in Table S3). ENSEMBL gene identifiers (ENSG accession numbers) and Reference Sequence mRNA identifiers (RefSeq; NM) were retrieved from the Clone IDs (IMAGE numbers) represented on the arrays using the CLONE|GENE ID converter (http://idconverter.bioinfo.cnio.es/) [68]. Replicate probes representing the same transcript were collapsed to ENSEMBL or RefSeq annotated transcripts ( = unique transcripts), which were then mapped to genes based on ENSG accession numbers ( = annotated genes). All microarray data is available at the Stanford Microarray Database (SMD) or at the Gene Expression Omnibus at www.ncbi.nlm.nih.gov/geo (GSE12357).

To compare our PUM1 mRNA targets with the ones defined by Morris *et al.* [61], we retrieved the ENSG and RefSeq accession numbers of the Morris *et al.* study from GEO (accession No. GSE 11301, platform GPL5770) and from the Supplemental Material published on the journal's web site.

### Synthesis of biotinylated RNAs and pull-down experiments

DNA templates for biotin-RNA synthesis were prepared by PCR from 200 ng of HeLa S3 genomic DNA with 5′-oligonucleotides bearing a T7 RNA polymerase promoter sequence, except for MET where complementary pairs of oligonucleotides comprising nts 1950–2006 of MET were annealed and cloned into psiCheck-2 (Promega). The following oligonucleotide pairs were used to amplify the indicated regions (specified by nucleotide positions) of 3′-UTRs: INTS2-T7Fw and INTS2-Rev for nucleotides (nts) 1800–2144 of INTS2, DCUN1D3-T7Fw and DCUN1D3-Rev for nts 965–1474 of DCUN1D3, Dll1-T7Fw and Dll1-Rev for nts 120–587 of Dll1, SDAD1-T7Fw and SDAD1-Rev for nts 112–529 of SDAD1, VEGFA-T7Fw and VEGFA-Rev for nts 925–1485 of VEGF-A. The ORF plus 500 nts downstream of the yeast *COX10* gene was amplified with primers COX10-T7Fw and COX10-Cnot from *S. cerevisiae* genomic DNA. The Rps26 control probe was prepared as described [36]. Biotinylated RNAs were produced with T7-RNA polymerase with biotin RNA labeling mixture (Roche) as described [36].

Biotin RNA pull-down experiments were performed essentially as described [36]. Extracts were prepared by mechanical disruption with a Tissue Lyser (Qiagen; 6× 30 sec, 30 Hz, 4°C) from HeLa S3 cells that were either transiently transfected with pcDNA3.1-PUM1-HD-TAP and collected after 24 hours, or that stably expressed PUM2-HD-TAP. 130 µg (protein content) of extract was incubated with 2 pmol of biotinylated RNAs, and streptavidin captured RNA-protein complexes were resolved on a 10% SDS polyacrylamid gel. Proteins were visualized with PAP antibody or specific anti-PUM antibodies.

### Web-based database searches

Protein Analysis THrough Evolutionary Relationships (PANTHER) analysis was performed with PUM1 and PUM2 mRNA targets (unique transcripts with 5% FDR) at http://www.pantherdb.org/ [46]. Gene Ontology (GO) searches were performed with the Generic Gene Ontology Term Finder (http://go.princeton.edu/cgi-bin/GOTermFinder) [47]. For comparative analysis of mRNA targets, ENSG IDs for predicted human orthologs of *Drosophila* Pum and *S. cerevisiae* Puf3p targets [35], [36] were retrieved with Biomart (http://www.biomart.org/) [69].

### Motif searches

3′-UTR, 5′-UTR and coding sequences were retrieved from ENSEMBL (via ENSG IDs; Ensembl Release 48/1st December 2007) or GenBank (via RefSeq; release 164/February 2008) [70], [71]. Motif searches were performed with MEME (http://meme.sdsc.edu/meme/meme.html) [51] on the first 100 3′-UTR sequences available corresponding to the 125 and 135 highest enriched (according to descending SAM score) PUM1 and PUM2 targets, respectively, with the following settings: searching the sense strand, one motif per sequence and 6 to 10 nucleotides expected motif length. The 3′-UTR, 5′-UTR and coding sequences of PUM1 and PUM2 targets (FDR<5%) were searched for PUF motifs (TGTAnATA) with PatSearch (http://www.ba.itb.cnr.it/BIG/PatSearch/) [52]

For the conservation analysis of PUF motifs in PUM1 and PUM2 targets and non-targets, the genomic location of PUF motifs found in PUM1 and PUM2 targets (IPed transcripts) and non-targets (expressed but not IPed transcripts) was inferred by aligning the mRNAs to the hg18 assembly of the human genome using the Spa algorithm [72], and the genomic coordinates of the PUF motif were identified based on the coordinates in the mRNA and the mRNA-to-genome alignments. The phastCons conservation scores for each nucleotide within 8 nucleotides-long regions centered on the middle of the PUF motifs were extracted from the UCSC site (http://hgdownload.cse.ucsc.edu/goldenPath/hg18/database/phastCons17way.txt.gz) [54]. For each position around the PUF motif we then constructed two vectors: one that contained the conservation scores for that particular position around PUF motifs in IPed transcripts, and the other containing the conservation scores for that position around PUF motifs in transcripts that were expressed but not IPed. Finally, we applied the Wilcoxon test to the two vectors of conservation scores and reported the position-wise profile of the logarithm of the *p*-value.

### Extraction of miRNA target sites

From http://www.mirz.unibas.ch/ElMMo2 we extracted miRNA target predictions generated based on the algorithm previously described [58]. We extracted as high-confidence target sites the top 1000 sites in the order of their posterior probability of being under functional selection. An equal number of low-confidence target sites was extracted by traversing the list of predicted sites for each miRNA from the sites with lowest probability to those with the highest probability, and selecting, for each miRNA a number of low-probability sites equal to the number of high-probability sites.

### Motif searches with the Phylogibbs algorithm

To identify binding sites for protein cofactors of the miRNA pathway, we applied the Phylogibbs algorithm [57] to the 400 nucleotide upstream and downstream regions of the high-confidence sites of three miRNAs, which had a few hundred high-confidence predicted targets (miR-30a – 210 upstream/208 downstream regions, miR-19 – 126 upstream/154 downstream regions and miR-137 – 153 upstream/131 downstream regions). The 3′-UTRs of the predicted miRNA targets were mapped to the hg18 assembly of the human genome using the Spa algorithm for mRNA-to-genome mapping [72]. The genomic locations of the miRNA target sites were identified based on the location of the target sites in the 3′-UTRs and the alignments of 3′-UTRs to genome. The genomic coordinates of the predicted sites were then used to extract alignments that covered 400 nucleotides upstream or downstream of the miRNA match in the following species: mouse - mm8 assembly, rhesus monkey - rheMac2 assembly, dog - canFam2 assembly, cow - bosTau2 assembly and horse - equCab1 assembly. The pair-wise genome alignments were obtained from the genome browser web site of the University of California of Santa Cruz (http://hgdownload.cse.ucsc.edu/goldenPath/hg18/vsX, where X is the corresponding assembly as given above). The orthologous regions were realigned using the T-coffee algorithm, and then submitted to Phylogibbs. Without trying to perform an exhaustive study, we used the following parameters: motif length (m) = 10, number of different motifs to infer (z) = 2, expected number of sites in a given set of sequences (y) = 120, order of the Markov model for background probabilities (N) = 3.

### Computation of the density of high-confidence miRNA targets in the 3′-UTRs of PUM1 and PUM2 targets and non-targets

We intersected the set of mRNAs that had at least one high-confidence (p≥0.5) predicted miRNA target site in their 3′-UTRs with the sets of mRNAs that were IPed, or expressed but not IPed in the PUM1 and PUM2 experiments. Then, for each mRNA, we computed the density of high-confidence targets sites per 3′-UTR nucleotide by dividing the number of high-confidence sites in the 3′-UTR by the total length of the 3′-UTR.

## Supporting Information

Text S1 Oligonucleotide primer sequences.
(0.03 MB DOC)

Figure S1 Immunoblot analysis of human PUM proteins in HeLa S3 cells. (A) Expression of endogenous human PUM proteins in HeLa S3 cells. Lane 1: Immunoblot analysis of PUM1 (127 kDa) probed with anti-PUM1 antibody (25 µg cell extract); lane 2: Immunoblot analysis of PUM2 (114 kDa) probed with anti-PUM2 antibody (50 µg cell extract). (B) Immunoblot analysis following immunoprecipitation of PUM1 and PUM2 with anti-PUM1 and anti-PUM2 antibodies. Lanes 1–4: PUM affinity isolations; lanes 5–8: mock control isolations. Lanes 1, 5: cell extract; lanes 2, 6: supernatant after incubation of extracts with antibody-coupled protein G or protein A sepharose beads; lanes 3, 7: RNP eluates after treatment of beads with SDS-EDTA; lanes 4, 8: RNP eluates probed with the alternate PUM antibody. 25 µg (PUM1) or 50 µg (PUM2) of extracts and supernatants, 5% of captured beads and 1% of eluates were loaded.
(3.61 MB TIF)

Table S1 mRNA specifically associated with PUM1 and PUM2. Columns indicate the following (from left to right): total number of transcripts (including replicates); total number of unique transcripts; total number of transcripts with ENSEMBL gene IDs; all listed according to FDRs determined by SAM.
(0.02 MB XLS)

Table S2 List of PUM1 target mRNAs in HeLa S3 cells. Columns indicate the following (from left to right): Clone_ID (IMAGE); gene name; gene description; average log2 ratio in PUM1 affinity isolations; average log2 ratio in mock affinity isolations; SAM score; FDR; Ensembl_Gene_ID; Ensembl_Gene (+); RefseqRNA; EntrezGene; GenBank accession number; PUM2 target (+); PUM2 affinity isolation FDR; 3′-UTR information available (+); 3′-UTR information available from ENSEMBL, ENSG (+); PUF motif within 3′-UTR (+); PUF motif within 3′-UTR from ENSG sequence (+); number of motifs within 3′-UTR; CDS information available (+); CDS information available from ENSEMBL, ENSG (+); PUF motif within CDS (+); PUF motif within CDS from ENSG sequence (+); number of motifs within CDS; 5′-UTR information available (+); 5′-UTR information available from ENSEMBL, ENSG (+); PUF motif within 5′-UTR (+); PUF motif within 5′-UTR from ENSG sequence (+); number of motifs within 5′-UTR; miRNA binding site close (within 50 nts) to 3′-UTR PUF motif (+); distance between PUF and miRNA sites (nts).
(0.78 MB XLS)

Table S3 List of PUM2 target mRNAs in HeLa S3 cells. Columns indicate the following (from left to right): Clone_ID (IMAGE); gene name; gene description; average log2 ratio PUM2 affinity isolations; average log2 ratio mock affinity isolations; SAM score; FDR; Ensembl_Gene_ID; Ensembl_Gene (+); RefseqRNA; EntrezGene; GenBank accession number; PUM1 target (+); PUM1 affinity isolation FDR; 3′-UTR information available (+); 3′-UTR information available from ENSEMBL, ENSG (+); PUF motif within 3′-UTR (+); PUF motif within 3′-UTR from ENSG sequence (+); number of motifs within 3′-UTR; CDS information available (+); CDS information available from ENSEMBL, ENSG (+); PUF motif within CDS (+); PUF motif within CDS from ENSG sequence (+); number of motifs within CDS; 5′-UTR information available (+); 5′-UTR information available from ENSEMBL, ENSG (+); PUF motif within 5′-UTR (+); number of motifs within 5′-UTR; PUF motif within 5′-UTR from ENSG sequence (+); miRNA binding site close (within 50 nts) to 3′-UTR PUF motif (+); distance between PUF and miRNA sites (nts).
(0.32 MB XLS)

Table S4 Significantly shared PANTHER and GO annotations among PUM1 and PUM2 mRNA targets. (A) Significantly shared PANTHER annotations among PUM1 mRNA targets. (B) Significantly shared PANTHER annotations among PUM2 mRNA targets (C) Significantly shared GO annotations among PUM1 targets (D) Significantly shared GO annotations among PUM2 targets.
(0.06 MB XLS)

Table S5 Conservation between yeast, Drosophila and human PUM targets. (A, B) Homologous messages conserved among yeast, Drosophila and human pumilio targets. (C) Significantly shared PANTHER annotations among 85 conserved PUM1 and Drosophila Pumilio mRNA targets.
(0.03 MB XLS)

Table S6 Statistics of PUF motif among PUM1 targets. (A) 3′-UTRs. (B) CDS. (C) 5′-UTR. Columns indicate the following (from left to right): search option; number of ENSEMBL genes; number of sequences retrieved from ENSEMBL; number of motifs (number of motifs from ENSEMBL-retrieved sequences); p-value.
(0.03 MB XLS)

Table S7 Statistics of PUF motif among PUM2 targets. (A) 3′-UTRs. (B) CDS. (C) 5′-UTR. Columns indicate the following (from left to right): search option; number of ENSEMBL genes; number of sequences retrieve from ENSEMBL; number of motifs (number of motifs from ENSEMBL-retrieved sequences); p-value.
(0.03 MB XLS)

Table S8 Motifs enriched in the surrounding of miRNA binding sites. Columns indicate the following: motif; number of positive sequences; number of negative sequences; p-value. (A) Motifs enriched downstream of miRNA binding sites. (B) Motifs enriched upstream of miRNA binding sites.
(1.22 MB XLS)

Table S9 List of PUM targets with conserved PUF and miRNA binding sites. Columns indicate the following: (A) Targets with PUF and miRNA conserved double sites among the species indicated in C (Homo sapiens, hg; Rhesus macaque, rheMac; Bos Taurus, bosTau; Canis familiaris, camFam; Mus musculus, mm). For each target, the first rows of C and D indicate the position of PUF binding sites (start-end); the first rows of F and G indicate the position of the miRNA binding sites (start-end) specified in E. H indicates the probability that the miRNA binding site is under selection; column I indicates the distance between PUM and miRNA binding sites.
(0.25 MB XLS)

Table S10 Significantly shared PANTHER and GO annotations among predicted human PUM targets. (A) Significantly shared PANTHER annotations among predicted human PUM targets. (B) Significantly shared GO annotations among predicted human PUM targets.
(0.05 MB XLS)
