# Supplementary material for: Comparative Analysis of mRNA Targets for Human PUF-Family Proteins Suggests Extensive Interaction with the miRNA Regulatory System
Source: PLoS One. 2008 Sep 8;3(9):e3164. doi: 10.1371/journal.pone.0003164 (PMC2522278; doi:10.1371/journal.pone.0003164)
Supplement: Text S1 — Oligonucleotide primer sequences. (0.03 MB DOC) [file pone.0003164.s001.doc]

**Supporting text (Galgano *et al.*)**

**Oligonucleotide primers.**

The following primers were used to generate PUM expression constructs (restriction sites are underlined):

TAP1-NotIFw: 5’-GATAGCGGCCGCCATGGAAAAGAGAAGATGG-3’

TAP2-XhoIRev: 5’-CTATCTCGAGCTTCAGGTTGACTTCCCCG-3’

PUM1-HD-EcoRVFw: 5’-GATAGATATCATGCCTCTCCCTAGTCAGG-3’

PUM1-HD-NotIRev: 5’-CTATGCGGCCGCGATGATACCATTAGGGGGGC-3’

PUM2-HD-EcoRVFw: 5’-GATAGATATCATGCCTCTGCCAAGCCAAAC-3’

PUM2-HD-NotIRev: 5’-CTATGCGGCCGCCAGCATTCCATTTGGTGGTC-3’

The following PCR primers were used to provide templates for *in vitro* transcription (T7 promoter in lower-case):

INTS2-T7Fw: 5’-taatacgactcactataggGGCAGTTTTGTAGTCCCTTAA-3’

INTS2-Rev: 5’-AGCTGCACACATCTGTTTTCAC 3’

DCUN1D3-T7Fw: 5’-taatacgactcactataggGGGAAACTGCCTGCCGGCCAAC-3’

DCUN1D3-Rev: 5’-ATTATGGCATTCCCTGGCACTC-3’

Dll1-T7Fw: 5’-taatacgactcactatagggagttcagaccgagcaggttc-3’

Dll1-Rev: 5’-CATTTGCACAATATTCCATAAATAC-3’

SDAD1-T7Fw: 5’-taatacgactcactatagggtgaaaactgctgaacatgtgg-3’

SDAD1-Rev: 5’-CAGCAGGAAAGCCTATGTACAAG-3’

VEGFA-T7Fw: 5’-taatacgactcactatagggtcccggcgaagagaagagac-3’

VEGFA-Rev: 5’-GGGAGGGCAGAGCTGAGTGTTA-3’

cox10-T7Fw: 5’- taatacgactcactatagggTGAGTTACTTTCCTCGAACATATG-3’

COX10-Cnot: 5’-CatgcggccgcGAAGAGGACATGGAAGCTATTGGG-3’

The following complementary oligonucleotide pairs were used for *in vitro* transcription of MET 3’-UTR fragments bearing wild-type or mutant PUM binding sites (underlined):

MET-WTFw: 5’-TCGAGCCCATCAACAGGACTACACACTTGTATATACATTCTTGAGAACACTGCGC-3’

MET-WTRev: 5’-GGCCGCGCAGTGTTCTCAAGAATGTATATACAAGTGTGTAGTCCTGTTGATGGGC-3’

MET-MUTFw: 5’- TCGAGCCCATCAACAGGACTACACACTACAATATACATTCTTGAGAACACTGCGC-3’

MET-MutRev: 5’-GGCCGCGCAGTGTTCTCAAGAATGTATATTGTAGTGTGTAGTCCTGTTGATGGGC-3’
