# Supplementary figures and images for: Comparative Analysis of mRNA Targets for Human PUF-Family Proteins Suggests Extensive Interaction with the miRNA Regulatory System
Source: PLoS One. 2008 Sep 8;3(9):e3164. doi: 10.1371/journal.pone.0003164 (PMC2522278; doi:10.1371/journal.pone.0003164)

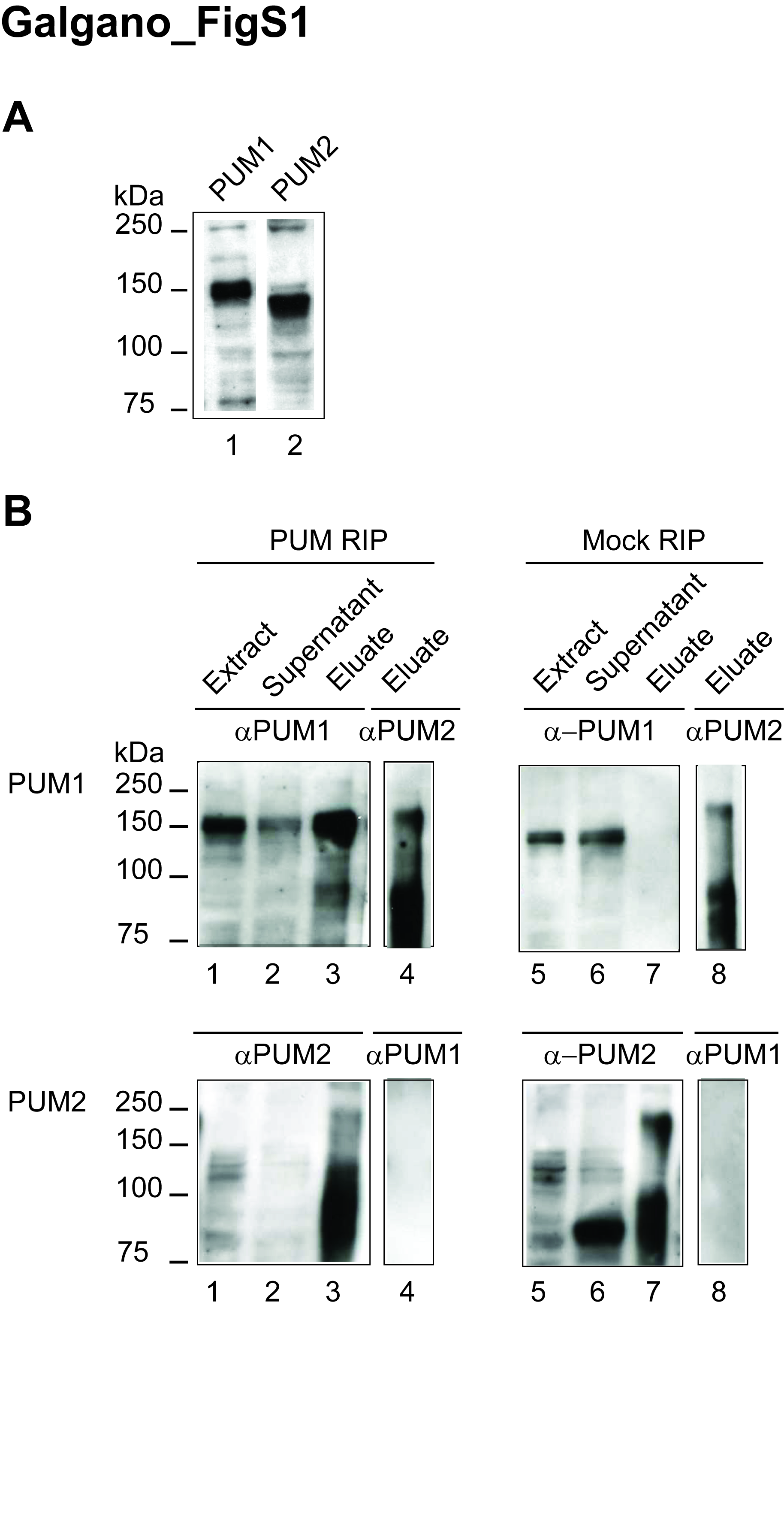

Supplement: Figure S1 — Immunoblot analysis of human PUM proteins in HeLa S3 cells. (A) Expression of endogenous human PUM proteins in HeLa S3 cells. Lane 1: Immunoblot analysis of PUM1 (127 kDa) probed with anti-PUM1 antibody (25 µg cell extract); lane 2: Immunoblot analysis of PUM2 (114 kDa) probed with anti-PUM2 antibody (50 µg cell extract). (B) Immunoblot analysis following immunoprecipitation of PUM1 and PUM2 with anti-PUM1 and anti-PUM2 antibodies. Lanes 1–4: PUM affinity isolations; lanes 5–8: mock control isolations. Lanes 1, 5: cell extract; lanes 2, 6: supernatant after incubation of extracts with antibody-coupled protein G or protein A sepharose beads; lanes 3, 7: RNP eluates after treatment of beads with SDS-EDTA; lanes 4, 8: RNP eluates probed with the alternate PUM antibody. 25 µg (PUM1) or 50 µg (PUM2) of extracts and supernatants, 5% of captured beads and 1% of eluates were loaded. (3.61 MB TIF) [file pone.0003164.s002.tif]
